# Circulating Fatty Acid Composition and Subsequent Adiposity Gain: A Prospective Study in the China Kadoorie Biobank

**DOI:** 10.3390/nu18203401

**Published:** 2026-10-16

**Authors:** Haozhi Niu, Menghan Wang, Sihong Wei, Guoqing Ma, Huiying Wang, Hongkai Xu, Yu Wang, Xin Liu

**Affiliations:** 1 Department of Epidemiology and Biostatistics, School of Public Health, Xi’an Jiaotong University, Xi’an 710061, China; 2 Key Laboratory for Disease Prevention and Control and Health Promotion of Shaanxi Province, Key Laboratory of Environment and Genes Related to Diseases of Ministry of Education of China, Xi’an Jiaotong University, Xi’an 710061, China

**Keywords:** fatty acids, fatty acid composition, adiposity gain, body mass index, longitudinal cohort, China Kadoorie Biobank

## Abstract

**Background/Objectives**: Evidence linking circulating fatty acids to adiposity gain remains limited. We examined associations of circulating fatty acid composition with longitudinal adiposity progression among Chinese adults. **Methods**: We included 1498 participants from the China Kadoorie Biobank with baseline nuclear magnetic resonance measurements of circulating fatty acid proportions and repeated measurements of body mass index (BMI) and waist circumference over a median of 8.22 years. Excessive adiposity increase was defined a priori as a BMI increase of ≥5% from baseline. Modified Poisson regression was used to estimate risk ratios (RRs) or prevalence ratios and their 95% confidence intervals (CIs) with multivariable adjustment when appropriate. **Results**: Overall, 499 participants (33.3%) experienced excessive adiposity increase. A one-standard-deviation (SD) higher monounsaturated fatty acid (MUFA) proportion was associated with a lower risk of excessive adiposity increase (RR = 0.870; 95% CI, 0.801–0.945; *q* = 0.004), whereas a higher ω-6 proportion was associated with a higher risk (RR = 1.115; 95% CI, 1.029–1.208; *q* = 0.016). However, in the cross-sectional analyses, MUFA and ω-6 showed associations with baseline overweight or obesity in directions opposite to their prospective associations with excessive adiposity increase. **Conclusions**: Higher circulating MUFA and ω-6 proportions were associated with lower and higher risks of a ≥5% BMI increase, respectively. The associations were less consistent across alternative outcome definitions, particularly for ω-6.

## 1. Introduction

Obesity is a growing global public health challenge, with its prevalence increasing markedly over recent decades. In 2021, approximately 2.11 billion adults aged 25 years or older worldwide were living with overweight or obesity [1]. Excess adiposity contributes substantially to the burden of cardiovascular disease, type 2 diabetes, metabolic syndrome, certain cancers, and premature mortality [2]. Identifying metabolic profiles that precede sustained adiposity gain may help clarify the processes involved in obesity development.

Adiposity progression involves adipocyte hypertrophy and hyperplasia, accompanied by altered adipokine secretion, immune-cell infiltration, impaired lipid storage, and adipose-tissue dysfunction [3,4]. In insulin-resistant adipose tissue, impaired suppression of lipolysis by insulin can increase fatty acid release into the circulation. Circulating fatty acid profiles reflect both dietary intake and endogenous biological processes, including de novo lipogenesis, fatty acid desaturation and elongation, adipose-tissue lipolysis, and lipoprotein metabolism [5,6]. Experimental evidence indicates that individual fatty acid classes can affect pathways involved in adiposity development. Oleic acid, a major monounsaturated fatty acid (MUFA), can enhance complete fatty acid oxidation through protein kinase A (PKA)–sirtuin 1 (SIRT1)–peroxisome proliferator-activated receptor γ coactivator-1α (PGC-1α) signalling in cultured C2C12 myotubes [7]. Arachidonic acid-derived mediators have differing effects on adipogenesis: prostacyclin can promote differentiation, whereas prostaglandin F2α can inhibit it. In 3T3-L1 preadipocytes, exposure to arachidonic acid during the first 24 h inhibited later differentiation through prostaglandin F2α–Fos-related antigen 1 signalling [8]. A randomized trial in severely obese adults without diabetes found that long-chain ω-3 supplementation reduced adipose-tissue and systemic inflammation, whereas experimental studies in rodents and isolated muscle linked saturated-fat-induced insulin resistance to ceramide synthesis [9,10]. These findings provide a biological basis for examining whether circulating fatty acid composition is associated with subsequent adiposity gain.

Previous studies have identified clear associations between circulating fatty acids and existing adiposity, but their temporal interpretation remains uncertain. In a large cross-sectional nuclear magnetic resonance (NMR) study, greater adiposity was associated with higher circulating concentrations of most fatty acids, with particularly strong associations for MUFA [11]. Plasma linoleic acid and total ω-6 fatty acid proportions, by contrast, were inversely associated with weight and waist circumference in the UK Biobank [12]. The relationship is potentially bidirectional: changes in fatty acid synthesis, desaturation, lipolysis, and lipid transport as adiposity develops may also alter circulating fatty acid composition. A circulating profile may therefore reflect consequences of existing adiposity as well as characteristics that precede subsequent gain. Prospective findings have also been inconsistent. Higher erythrocyte ω-6 fatty acids and the ω-6-to-ω-3 ratio were associated with greater 10-year weight gain among initially normal-weight women [13], whereas the Multi-Ethnic Study of Atherosclerosis found no clear associations of plasma phospholipid ω-3 or ω-6 fatty acids with subsequent body mass index (BMI) change [14]. A 2026 UK Biobank study reported inverse longitudinal associations for linoleic acid but positive associations for non-linoleic ω-6 fatty acids, indicating that associations may differ among fatty acid species [12]. Prospective evidence has therefore focused mainly on selected polyunsaturated fatty acid (PUFA) species and Western populations, and it remains uncertain whether baseline proportions of the major circulating fatty acid classes are associated with subsequent BMI gain after accounting for baseline BMI in Chinese adults, and how these associations compare with those observed for existing adiposity. BMI is widely used to assess general adiposity, whereas waist circumference (WC) provides a complementary measure of abdominal adiposity and related cardiometabolic risk [15].

Using baseline and second-resurvey data from the China Kadoorie Biobank (CKB), we examined whether the circulating proportions of saturated fatty acids (SFA), MUFA, ω-6, and ω-3 were associated with the risk of excessive adiposity increase (a ≥5% increase in BMI) over approximately 8 years after adjustment for baseline BMI. We also compared these prospective associations with cross-sectional associations with baseline overweight or obesity. Associations with WC progression were evaluated as complementary analyses.

## 2. Materials and Methods

### 2.1. Study Population and Design

The CKB is a prospective cohort study whose design and methodology have been described in detail elsewhere [16,17]. Between June 2004 and July 2008, 512,891 adults aged 30–79 years were recruited from ten geographically diverse study regions across China, comprising five rural and five urban areas with varying socioeconomic circumstances. The first and second resurveys were conducted between June and October 2008 and between August 2013 and September 2014, respectively. Each resurvey included approximately 5% of surviving participants selected at random. At baseline and each resurvey, trained health professionals administered electronic questionnaires to collect data on sociodemographic characteristics, lifestyle factors, medical history, and medication use. Standardized anthropometric measurements were obtained, and a 10 mL non-fasting blood sample was collected for long-term storage and subsequent analyses.

The present analysis drew on CKB participants with baseline NMR metabolomics data who attended the second resurvey. Participants had been selected for baseline NMR profiling through several sampling frames within the CKB, including a nested case–control study of incident myocardial infarction, ischemic stroke, and intracerebral hemorrhage [18], second-resurvey sampling, and a pancreatic cancer and diabetes study. Among 1515 participants with baseline NMR metabolomics data who attended the second resurvey, 1502 had BMI measurements at both baseline and the second resurvey. We further excluded four participants whose fatty acid measurements did not meet the NMR quality-control criteria, leaving 1498 participants for analysis. Each participant contributed one observation to the analysis, including those selected through more than one NMR study. Sampling-source membership and overlap are summarized in Table S1. Ethical approval for the China Kadoorie Biobank was obtained from the Ethical Review Committee of the Chinese Center for Disease Control and Prevention (approval number: 005/2004; approval date: 11 May 2004) and the Oxford Tropical Research Ethics Committee, University of Oxford (approval number: 025-04; approval date: 3 February 2005). All participants provided written informed consent. The present analysis was conducted under an approved CKB data-access application.

### 2.2. Anthropometric Measurements

Anthropometric measurements were obtained at baseline and during resurveys using standardized protocols and calibrated equipment. Height was measured to the nearest 0.1 cm with a stadiometer, with participants standing barefoot. Body weight was recorded to the nearest 0.1 kg using a Tanita TBF-300 Body Composition Analyzer (Tanita Inc., Tokyo, Japan), accounting for clothing weight (0.5 kg in summer, 1.0 kg in spring/autumn, and 2.0–2.5 kg in winter). WC was measured to the nearest 0.1 cm using a non-elastic tape placed midway between the lowest rib and the iliac crest. BMI was calculated as weight (kg) divided by height squared (m^2^). Excessive adiposity increase was defined a priori as a BMI increase of ≥5% from baseline. This operational definition was consistent with a previous study [19]. Alternative thresholds and continuous measures of change were examined in additional analyses.

### 2.3. Fatty Acid Quantification

At baseline, blood samples were centrifuged and plasma was aliquoted at regional laboratories before transport to Oxford, United Kingdom, for long-term storage. They were later thawed and sub-aliquoted at the Wolfson Laboratory (University of Oxford, Oxford, UK), with 100 μL plasma aliquots analyzed using NMR spectroscopy at the Brainshake Laboratory (Kuopio, Finland) [18]. A high-throughput targeted NMR metabolomics platform was used to quantify 225 lipid and metabolic measures, including the relative proportions of circulating fatty acids [6]. This study focused on the proportions of SFA, MUFA, ω-3 PUFA, and ω-6 PUFA. For the primary exposure analyses, each fatty acid class was expressed as a percentage of total circulating fatty acids rather than as an absolute concentration. Analytical reliability was previously assessed by Holmes et al. using duplicate NMR measurements of 137 CKB samples. The reported coefficients of variation for the proportions of SFA, MUFA, ω-6, and ω-3 were 2.23%, 2.68%, 1.89%, and 2.95%, respectively [18]. For the present analysis, we used the quality-control flags provided with the CKB NMR data. Participants were retained only when all nine flags for total fatty acids, SFA, MUFA, PUFA, ω-6, ω-3, linoleic acid, docosahexaenoic acid (DHA), and degree of unsaturation were nonmissing and coded 0, indicating no flagged quality-control issues. Four participants did not meet this criterion and were excluded.

### 2.4. Statistical Analyses

Baseline characteristics were summarized as unadjusted means and standard deviations (SDs) or numbers (percentages). Percentages were calculated using the nonmissing denominator for each variable. Within the analytic sample, there were no missing values in the fatty acid measures, anthropometric measurements or covariates used in the primary models. Sample sizes are reported with the corresponding analyses. Analyses used complete cases for the variables required by each model, without imputation. SFA, MUFA, ω-6, and ω-3 proportions were standardized to z-scores using their means and SDs in the full analytic sample, with the same exposure scale retained in restricted analyses, and associations were reported per 1-SD higher fatty acid proportion. The SDs and Pearson and Spearman correlations are provided in Table S2. The primary outcome, excessive adiposity increase, was defined as a ≥5% increase in BMI from baseline, while a ≥5% increase in WC was examined as a secondary outcome.

Covariates included age; sex; study region as a 10-category variable; education (no formal school, primary school, middle school, high school, college, or university); household income (<2500, 2500–4999, 5000–9999, 10,000–19,999, 20,000–34,999, or ≥35,000 Chinese yuan [CNY]/year); marital status (married, divorced, widowed, or never married); smoking status (never, occasional, ex-regular, or current regular); alcohol consumption (never, ex-regular, occasional, monthly, reduced intake, or weekly); physical activity (metabolic equivalent of task hours per day [MET-h/d]); and consumption frequencies of rice, wheat, other staple foods, meat, poultry, fish, and eggs. Total physical activity was calculated by multiplying the time spent on each activity by its corresponding metabolic equivalent value, summing across activities, and expressing the total as MET-h/d. The dietary frequency variables were included to reduce potential confounding by habitual food consumption but were not used to estimate energy, total fat, or individual fatty acid intake. All longitudinal models were additionally adjusted for the corresponding baseline adiposity measure. Each individual fatty acid proportion was evaluated in a separate model. Single-exposure models describe associations with the relative proportion of each fatty acid class, without specifying which other fatty acids decrease as that proportion increases.

For the primary longitudinal analyses, modified Poisson regression with a log link and heteroscedasticity-consistent type 0 robust variance estimation was used to estimate risk ratios (RRs) and 95% confidence intervals (CIs) for excessive adiposity increase [20]. Associations were first estimated per 1-SD higher fatty acid proportion. Fatty acid proportions were also categorized into quartiles, and quartile-specific RRs and floating 95% CIs were calculated using the floating absolute risk method, which permits comparisons between any two quartiles [21]. Linear trends were assessed by assigning participants the median fatty acid value of their quartile and modelling this value as a continuous variable. Dose–response relationships were further examined using restricted cubic splines with four knots placed at the 5th, 35th, 65th, and 95th percentiles of the exposure distribution, with the median exposure as the reference. The overall *p* value tested the joint contribution of all spline terms, whereas the *p* value for nonlinearity tested the nonlinear spline terms.

For the continuous longitudinal outcome, analysis of covariance (ANCOVA) was used, with follow-up BMI as the dependent variable and baseline BMI as a covariate. β coefficients and 95% CIs therefore represent the difference in follow-up BMI (kg/m^2^) associated with a 1-SD higher baseline fatty acid proportion, conditional on baseline adiposity and other covariates. Heteroscedasticity-consistent type 3 (HC3) standard errors were used in all linear regression models. Modified Poisson regression with robust variance was used to estimate prevalence ratios (PRs) and 95% CIs for baseline overweight or obesity (BMI ≥ 24 kg/m^2^).

Secondary analyses examined two log-ratio measures derived from circulating fatty acid concentrations using the isometric log-ratio balance formulation [22]. The first contrasted MUFA with the geometric mean of ω-6 and ω-3 and was calculated as √(2/3) × ln [MUFA/√(ω-6 × ω-3)]. The second contrasted ω-6 with ω-3 and was calculated as √(1/2) × ln(ω-6/ω-3). Both measures were standardized before analysis. Models for these ratios were additionally adjusted for log-transformed total circulating fatty acid concentration.

Compositional reallocation analyses evaluated a 1-percentage-point decrease in one fatty acid proportion accompanied by an equal increase in another among SFA, MUFA, ω-6, and ω-3, while the other two proportions remained constant. The four proportions were first rescaled to sum to 100%. For each contrast, the component being decreased was omitted from the model, and the other three components were included together with baseline BMI, standardized log-transformed total circulating fatty acid concentration, and all covariates listed above. The coefficient for the component being increased therefore represented the corresponding 1-percentage-point reallocation. All six pairwise reallocations were examined using modified Poisson regression for excessive adiposity increase and ANCOVA for follow-up BMI. These estimates describe contrasts within circulating fatty acid composition rather than effects of dietary substitution.

Additional outcome analyses examined absolute and annualized BMI changes using linear models with HC3 robust standard errors. Annualized change was calculated by dividing absolute BMI change by the interval between measurements, estimated from the difference in recorded age. Among participants with baseline BMI < 24 kg/m^2^, modified Poisson regression assessed overweight or obesity at the second resurvey, defined as BMI ≥ 24 kg/m^2^. All models included baseline BMI and the original covariates. The incident-overweight model additionally included the measurement interval (Table S3).

Sensitivity analyses repeated the primary modified Poisson models using BMI increase thresholds of ≥3% and ≥10%, after excluding participants selected as disease cases in the contributing NMR studies, or after additional adjustment for or stratification by sampling source. We also repeated the main analyses after excluding participants with baseline diabetes, chronic heart disease, stroke or transient ischemic attack, or cancer (Table S4). Sampling weights were not applied because the probabilities of selection into the contributing studies and participation in the second resurvey were unavailable. Baseline diabetes was defined as self-reported physician-diagnosed diabetes or screen-detected diabetes (fasting blood glucose ≥7.0 mmol/L or random blood glucose ≥11.1 mmol/L). Chronic heart disease, stroke or transient ischemic attack, and cancer were identified from self-reported physician diagnoses. Participants with unknown required disease status were excluded from the corresponding restricted analyses. Baseline disease and statin-use information is summarized in Table S5.

Subgroup analyses were performed for BMI increases of ≥5% according to median age (<45.6 or ≥45.6 years), sex, urban or rural area, education (primary school or below or middle school or above), household income (<20,000 or ≥20,000 CNY/year), current smoking, weekly alcohol consumption, median physical activity (<19.3 or ≥19.3 MET-h/d), and baseline BMI (<24 or ≥24 kg/m^2^). Models for the urban–rural subgroup analysis retained adjustment for the 10 CKB study regions. Effect modification was assessed by adding a multiplicative interaction term between each standardized fatty acid proportion and the subgroup variable.

All tests were two-sided. The false discovery rate (FDR) was controlled using the Benjamini–Hochberg procedure, with the correction families specified in Table S6. Results with *p* < 0.05 and *q* < 0.10 were considered statistically significant. All analyses were performed using R version 4.5.1.

## 3. Results

### 3.1. Baseline Characteristics

At baseline, the mean (SD) age was 47.9 (9.7) years, and 59.7% of participants were women. Over a median follow-up of 8.22 years (interquartile range, 7.61–8.81), 499 participants (33.3%) experienced a ≥5% increase in BMI. Participants with a ≥5% increase in BMI were younger and had lower baseline BMI and WC than those with an increase of <5% (Table 1).

### 3.2. Associations of Circulating Fatty Acid Proportions with the Risk of Longitudinal Adiposity Progression

**Figure 1:**
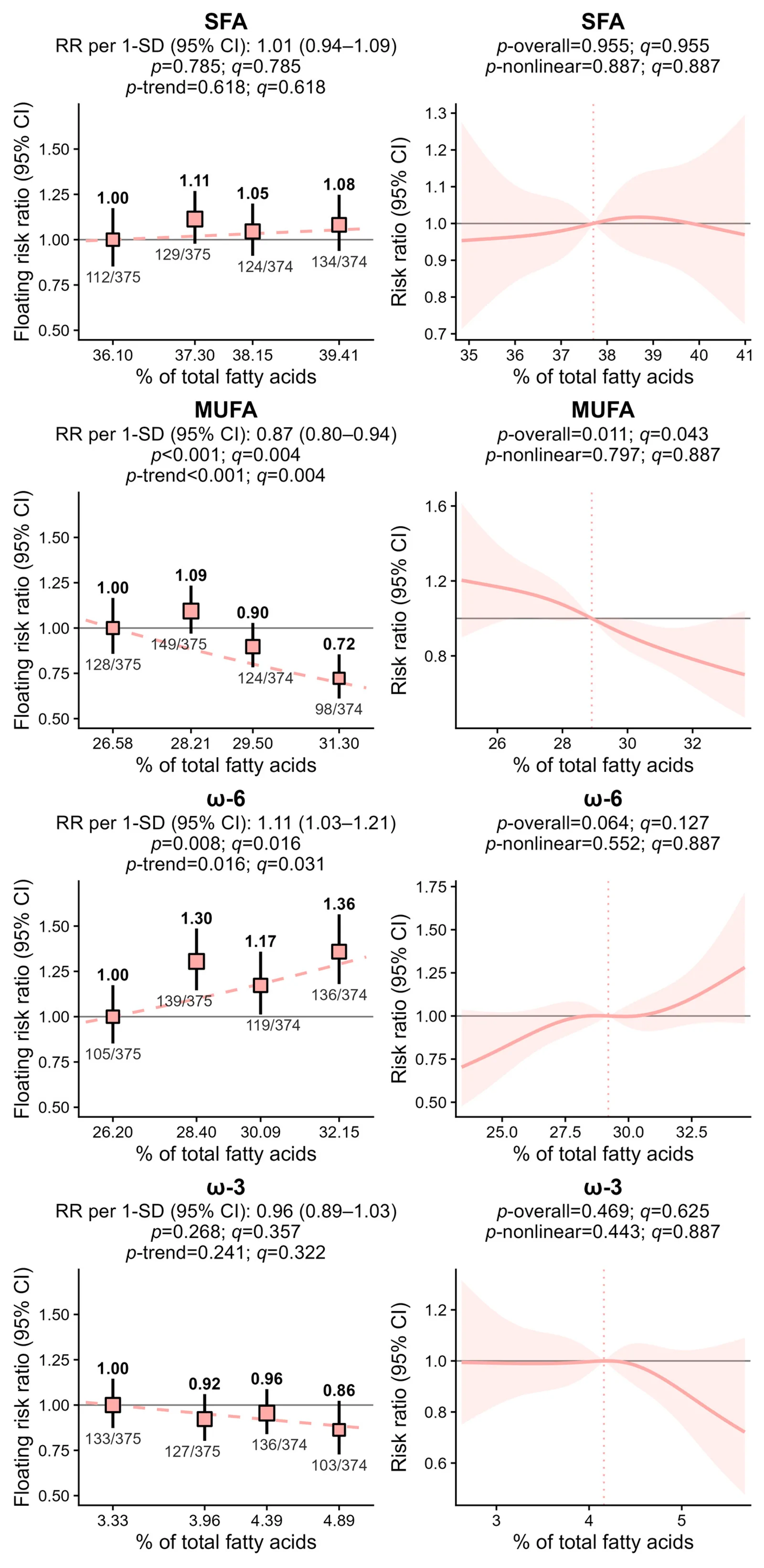
Excessive adiposity increase was defined as a ≥5% increase in BMI from baseline. The left column presents quartile-specific associations, and the right column presents restricted cubic spline analyses. Pink symbols and curves indicate the BMI associations. In the left column, squares and vertical lines represent floating RRs and 95% floating CIs at the median fatty acid proportion within each quartile; square size is proportional to floating precision. Numbers above the squares are RRs, and numbers below are events/participants. Dashed lines show the fitted log-linear associations obtained by modelling the quartile-specific median values continuously, with the median of the lowest quartile as the reference. In the right column, solid lines and shaded areas represent RRs and robust 95% CIs, and dotted vertical lines indicate the median exposure used as the reference. Restricted cubic spline models used four knots at the 5th, 35th, 65th, and 95th percentiles. Modified Poisson models with robust variance were adjusted for age, sex, the 10 CKB study regions, education, household income, marital status, smoking status, alcohol consumption, physical activity, dietary frequencies, and baseline BMI. The Benjamini–Hochberg procedure was applied across the four fatty acids separately for each type of statistical test. Confidence intervals were not adjusted for multiple testing. BMI, body mass index; CI, confidence interval; MUFA, monounsaturated fatty acids; RR, risk ratio; SD, standard deviation; SFA, saturated fatty acids; ω-3, omega-3 polyunsaturated fatty acids; ω-6, omega-6 polyunsaturated fatty acids.

After multivariable adjustment, a 1-SD higher MUFA proportion was associated with a lower risk of excessive adiposity increase (RR, 0.870; 95% CI, 0.801–0.945; *q* = 0.004), whereas a 1-SD higher ω-6 proportion was associated with a higher risk (RR, 1.115; 95% CI, 1.029–1.208; *q* = 0.016) (Figure 1). In the quartile analyses, the RRs of excessive adiposity increase in the highest quartile were 0.72 (95% CI, 0.61–0.85) for MUFA and 1.36 (95% CI, 1.18–1.56) for ω-6. The corresponding *q* values for trend were 0.004 and 0.031, respectively. No statistically significant association was observed for either SFA or ω-3. Apparent nonlinearity was not observed in the restricted cubic spline analyses (Figure 1).

When assessing the associations of baseline fatty acids with follow-up BMI, we observed that a 1-SD higher baseline MUFA proportion was associated with 0.119 kg/m^2^ lower follow-up BMI after multivariable adjustment, including baseline BMI (95% CI, −0.221 to −0.018; *q* = 0.086), whereas ω-6 was not significantly associated with follow-up BMI (Table S3A).

As a secondary analysis, we examined the associations between fatty acid proportions and long-term excessive WC increase (defined as a ≥5% increase) (Figure S1). No individual fatty acid proportion was significantly associated with excessive WC increase.

### 3.3. Associations with BMI Change and Incident Overweight or Obesity

For annualized BMI change, the estimates per SD were −0.014 kg/m^2^/year (95% CI, −0.027 to −0.002; *q* = 0.107) for MUFA and 0.009 kg/m^2^/year (95% CI, −0.004 to 0.021; *q* = 0.387) for ω-6. Neither association met the FDR criterion. Among participants with baseline BMI < 24 kg/m^2^, the RRs for overweight or obesity at resurvey, adjusted for baseline BMI, the original covariates, and the measurement interval, were 1.011 (95% CI, 0.885–1.156; *q* = 0.869) for MUFA and 0.914 (95% CI, 0.795–1.050; *q* = 0.410) for ω-6 (Table S3B–D).

### 3.4. Subgroup Analysis

**Figure 2:**
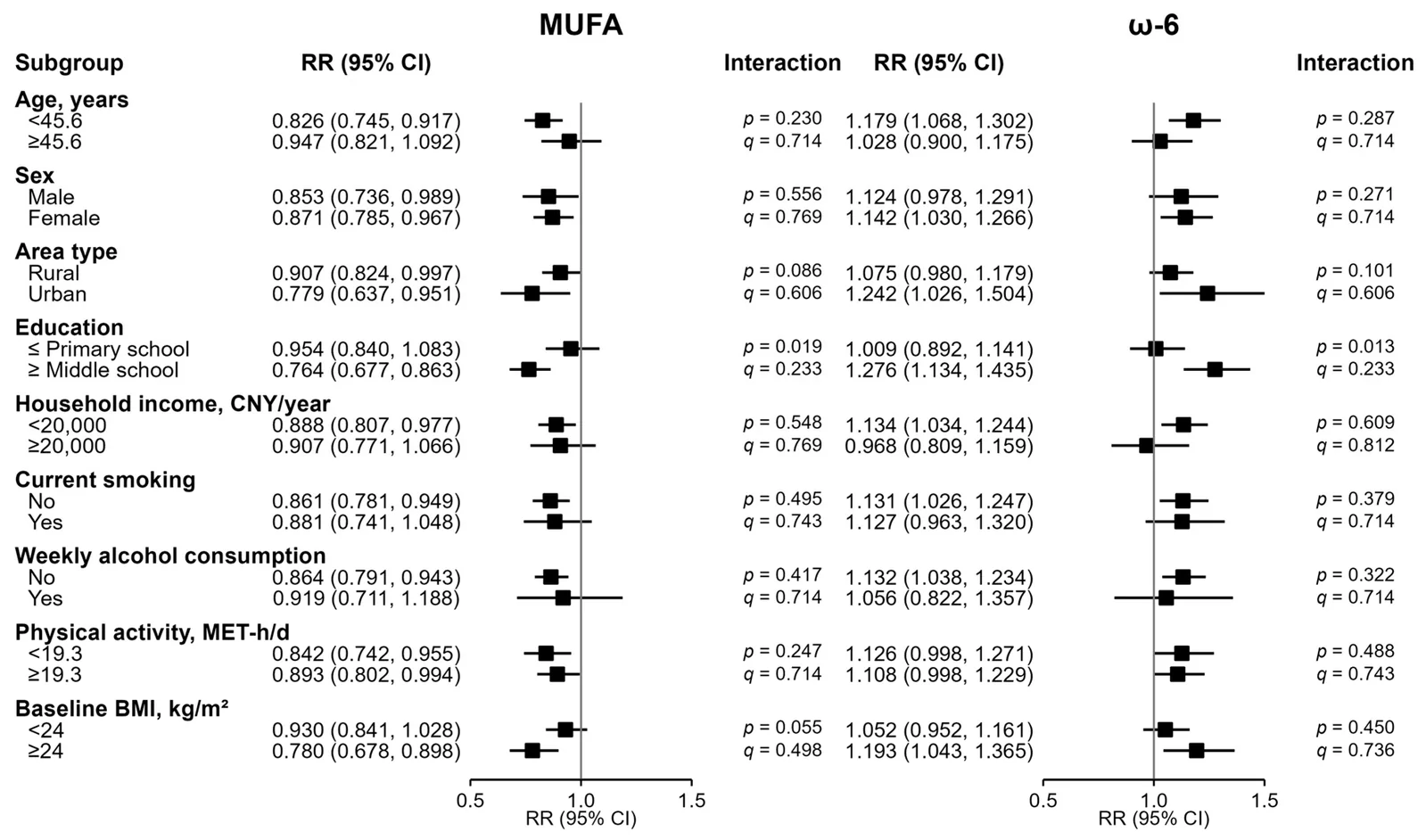
Excessive adiposity increase was defined as a ≥5% increase in BMI from baseline. RRs and 95% CIs are presented per 1-SD higher circulating MUFA or ω-6 proportion within each prespecified subgroup. Modified Poisson models with robust variance were adjusted for age, sex, the 10 CKB study regions, education, household income, marital status, smoking status, alcohol consumption, physical activity, dietary frequencies, and baseline BMI, excluding the stratification variable when appropriate. Squares and horizontal lines represent RRs and 95% CIs. Interaction *p* values were obtained by including a product term between the continuous fatty acid exposure and the subgroup variable. The displayed *q* values were calculated using Benjamini–Hochberg correction across all 36 interaction tests. Subgroup-specific confidence intervals were not adjusted for multiple testing. These analyses were exploratory. Participant and event counts for each subgroup are reported in Table S7B. BMI, body mass index; CI, confidence interval; CNY, Chinese yuan; MET-h/d, metabolic equivalent of task hours per day; MUFA, monounsaturated fatty acids; RR, risk ratio; SD, standard deviation; ω-6, omega-6 polyunsaturated fatty acids.

Associations were broadly consistent across the prespecified subgroups (Figure 2). None of the 36 interaction tests met the FDR criterion after joint correction (Table S7).

### 3.5. Sensitivity Analysis

When the BMI increase threshold was set at 3%, the inverse association with MUFA remained (RR per SD, 0.919; 95% CI, 0.862–0.981; *q* = 0.044), whereas the association with ω-6 did not meet the FDR criterion (RR, 1.061; 95% CI, 0.996–1.132; *q* = 0.134). Neither association met the FDR criterion at the 10% threshold. After excluding participants selected as disease cases in the contributing NMR studies, the associations with a ≥5% BMI increase remained for MUFA (RR, 0.879; 95% CI, 0.806–0.960; *q* = 0.016) and ω-6 (RR, 1.121; 95% CI, 1.030–1.219; *q* = 0.016). Additional adjustment for sampling source yielded corresponding RRs of 0.864 (95% CI, 0.792–0.942; *q* = 0.004) and 1.128 (95% CI, 1.033–1.232; *q* = 0.014), with directionally consistent estimates across the three sampling-source groups. After excluding participants with baseline diabetes, chronic heart disease, stroke or transient ischemic attack, or cancer, the corresponding RRs were 0.872 (95% CI, 0.802–0.948; *q* = 0.005) and 1.121 (95% CI, 1.033–1.216; *q* = 0.012) (Table S4).

### 3.6. Compositional Reallocation Analysis

Reallocating 1 percentage point from ω-6 to MUFA was associated with a lower risk of excessive adiposity increase (RR, 0.947; 95% CI, 0.909–0.986; *q* = 0.052). No other pairwise reallocation met the prespecified FDR criterion (Table S8).

### 3.7. Associations of Fatty Acid Log-Ratio Measures with Excessive Adiposity Increase

For the log-ratio measure comparing MUFA with the geometric mean of ω-6 and ω-3, the RR per SD was 0.920 (95% CI, 0.847–1.000), but the association did not meet the FDR criterion. Neither log-ratio measure met this criterion in the per-SD, quartile-trend, or spline analyses after joint correction across the ten BMI log-ratio tests (Figure S2; Table S9).

### 3.8. Associations of Fatty Acid Proportions with Baseline Overweight or Obesity and Excessive Adiposity Increase

A higher MUFA proportion was associated with a higher prevalence of baseline overweight or obesity (PR per 1-SD, 1.361; 95% CI, 1.287–1.440), despite its inverse association with a subsequent ≥5% BMI increase. Conversely, a higher ω-6 proportion was associated with a lower prevalence of baseline overweight or obesity (PR, 0.776; 95% CI, 0.735–0.819), while showing a positive association with a subsequent ≥5% BMI increase (Table 2).

## 4. Discussion

In this prospective study of 1498 Chinese adults followed for approximately 8 years, a higher baseline circulating MUFA proportion was associated with a lower risk of excessive adiposity increase, whereas a higher ω-6 proportion was associated with a higher risk. The quartile analyses supported both associations, and the spline analysis showed no evidence of nonlinearity. The baseline-adjusted association between MUFA and follow-up BMI was consistent in direction with the primary finding. No statistically significant association was observed between any individual fatty acid proportion and excessive WC increase. Cross-sectional analyses showed contrasting patterns, with MUFA positively and ω-6 inversely associated with baseline overweight or obesity.

Previous studies have linked MUFAs to several aspects of fatty acid oxidation and storage that may be relevant to adiposity progression. Oleic acid, a major MUFA, can activate PKA-dependent SIRT1-PGC-1α signalling and promote complete fatty acid oxidation [7]. Oleate can also facilitate the incorporation of saturated fatty acids into neutral triglyceride stores, thereby reducing the accumulation of lipotoxic intermediates and cellular apoptosis [23]. Consistent with this lipid-buffering model, diacylglycerol O-acyltransferase 1-mediated re-esterification of fatty acids in adipocytes protects the endoplasmic reticulum from lipotoxic stress and associated inflammatory responses during lipolysis [24]. However, circulating MUFA also reflects endogenous synthesis by stearoyl-CoA desaturase-1, an enzyme involved in lipogenesis, triglyceride synthesis, and very-low-density lipoprotein secretion. Previous studies have found that the relationships of stearoyl-CoA desaturase-1 with obesity and insulin resistance differ across the liver, visceral adipose tissue, and subcutaneous adipose tissue [25,26]. These findings provide biological context for the observed association. However, the relevant pathways were not measured in this study, and their contribution cannot be determined from circulating MUFA proportions alone.

When the BMI increase threshold was lowered to 3% or follow-up BMI was analyzed as a continuous outcome, the ω-6 estimates remained positive, but neither association met the FDR criterion. The association also did not meet the FDR criterion at the 10% threshold. Threshold-based analyses identify participants exceeding a specified relative increase, whereas continuous models assess differences across the outcome distribution. Changing the threshold also changes the number and characteristics of participants classified as events; the smaller number of events at the 10% threshold may reduce precision. These differences may contribute to the variation in results, although they do not establish a biological threshold or fully explain the findings. The circulating ω-6 pool includes linoleic acid and longer-chain products such as dihomo-γ-linolenic and arachidonic acids, whose formation depends on elongase and fatty acid desaturase (FADS) activity. These fatty acids give rise to lipid mediators with heterogeneous and sometimes opposing biological functions. Arachidonic acid-derived prostacyclin has been shown to promote preadipocyte differentiation through cyclic adenosine monophosphate-dependent signalling [27]. In an observational study, arachidonic acid-derived 5- and 11-hydroxyeicosatetraenoic acids were positively associated with BMI and WC, whereas vicinal diols derived from linoleic acid, eicosapentaenoic acid, and DHA showed inverse associations [28]. Evidence from randomized controlled trials indicates that increasing linoleic acid intake does not consistently increase conventional inflammatory markers [29], further supporting the biological heterogeneity within the ω-6 class. Genetic variation in FADS1 and FADS2 may additionally influence PUFA metabolism and adipose-tissue inflammatory signalling [30]. The present analysis used the combined ω-6 proportion rather than examining individual ω-6 species or downstream oxylipins. The result should therefore be interpreted as an association with the ≥5% BMI endpoint that requires further evaluation, rather than a general association with adiposity progression.

The analyses of fatty acid log-ratio measures and compositional reallocations provided additional information on the relative distribution of circulating fatty acid classes. The relative abundance of MUFA and PUFA has been examined in plasma lipidomics [31]. In this study, a higher MUFA-relative-to-PUFA log-ratio was nominally associated with a lower risk of excessive adiposity increase, but did not meet the FDR criterion. Reallocation from ω-6 to MUFA was associated with a lower risk of excessive adiposity increase, consistent with the contrasting associations observed for the individual MUFA and ω-6 proportions. These results also illustrate the interdependence of circulating fatty acid components [32,33]. The reallocation estimates describe contrasts within circulating composition and should not be interpreted as effects of replacing one dietary fatty acid with another.

The BMI findings were not consistently supported by the WC analyses. Subcutaneous adipose tissue constitutes the principal reservoir for whole-body triglyceride storage and buffers excess circulating fatty acids through adipocyte hypertrophy and recruitment. By contrast, visceral adipose tissue is more lipolytically active and releases fatty acids more readily into the portal circulation, thereby exerting pronounced effects on hepatic lipid metabolism and insulin sensitivity [34,35]. BMI reflects body mass relative to height without distinguishing fat from lean tissue, whereas WC reflects abdominal size without separating visceral and subcutaneous fat [15]. These differences support considering the two outcomes separately, but differences in statistical significance do not establish distinct biological effects. Without direct measurements of body composition and fat distribution, the findings cannot be attributed to a particular adipose-tissue compartment.

The contrasting cross-sectional and prospective associations should be interpreted in view of the different temporal questions addressed by these analyses. Circulating fatty acid profiles integrate dietary exposure with endogenous processes, including de novo lipogenesis, desaturation and elongation, adipose-tissue lipolysis, and hepatic lipid transport [36,37]. Consequently, the profile measured at a single time point may reflect both the metabolic consequences of existing adiposity and metabolic characteristics relevant to subsequent adiposity gain. Large NMR studies have similarly shown that greater adiposity is strongly associated with higher circulating MUFA concentrations and broad changes in systemic metabolism [11]. Longitudinal and Mendelian randomization evidence further indicates that greater adiposity can lead to extensive alterations in circulating fatty acids and other metabolites [38]. In this context, the positive cross-sectional association between MUFA and baseline adiposity may reflect enhanced endogenous desaturation or lipid turnover in response to existing fat accumulation. By contrast, the prospective analysis assessed whether baseline fatty acid composition preceded subsequent excessive adiposity increase. The opposing cross-sectional and prospective associations therefore need not represent conflicting findings. For MUFA, the annualized-change estimate was directionally consistent with the primary finding but did not meet the FDR criterion. The incident-overweight analysis provided limited evidence for these associations. These results indicate that the findings were less consistent across alternative outcome definitions.

Circulating fatty acid profiles are related to habitual dietary fat intake and may complement questionnaire-based dietary assessments by providing objective information on fatty acid exposure [39]. A recent dietary intervention study further indicated that changes in dietary fat quality are reflected in the circulating lipidome and may help characterize variation in cardiometabolic responses [40]. Individual differences in dietary fatty acid storage and oxidation have also been associated with long-term weight change in women [41]. Although that study measured tracer-derived fatty acid handling rather than circulating proportions, it supports further investigation of individual differences in fatty acid metabolism. Combining repeated fatty acid profiling with detailed dietary assessment could therefore help characterize individual metabolic responses to dietary fat composition. Controlled dietary studies incorporating species-specific fatty acid measurements and repeated assessments of adiposity are warranted to clarify the dietary determinants of these profiles and evaluate their potential value in nutritional interventions. Detailed energy-intake assessment, oxylipin measurements, and direct measures of body composition would help distinguish dietary exposure, endogenous metabolism, and changes in fat distribution.

The strengths of this study include its prospective design and repeated anthropometric assessments over approximately 8 years. Examining baseline overweight or obesity and subsequent adiposity progression in the same participants allowed us to distinguish fatty acid profiles associated with existing adiposity from those associated with subsequent change. Standardized anthropometric protocols and quantitative fatty acid measurements using a high-throughput NMR platform helped ensure measurement quality. The findings were further evaluated using continuous and binary outcomes, quartile and spline analyses, and sensitivity analyses using alternative outcome thresholds and excluding participants selected as disease cases, together with additional analyses addressing measurement interval, baseline disease, and sampling source. However, several limitations should also be considered. First, fatty acids were measured once in non-fasting plasma, and the analyses focused on broad fatty acid classes rather than individual species, lipid fractions, or downstream mediators. Within-person variation and limited molecular resolution may therefore have attenuated more specific associations. Although published duplicate measurements provide evidence of analytical repeatability, they do not characterize long-term within-person stability. Assay-batch identifiers and time since the last meal were unavailable in the data used for this study, preventing corresponding adjustment or sensitivity analyses. Second, dietary information was limited to consumption frequencies for selected food groups. Detailed information on energy intake, dietary fatty acid intake, cooking oils, and nutritional supplement use was unavailable, precluding direct assessment of the dietary determinants of circulating fatty acid profiles. Third, BMI and WC were measured at only two time points, precluding reconstruction of adiposity trajectories between surveys. Percentage change and the ≥5% threshold may therefore have been affected by measurement error, regression to the mean, and changes occurring between surveys. The ≥5% threshold was an operational definition, and its clinical significance over approximately eight years has not been established. The observed associations were modest in magnitude. BMI and WC are indirect measures that cannot distinguish changes in fat mass, lean mass, or individual fat depots. Measurement error in WC may also have attenuated its longitudinal associations. Fourth, the modest sample size limited statistical power, particularly for nonlinear and subgroup analyses, and residual confounding and reverse causation cannot be excluded. Participants with NMR measurements were drawn from several CKB sampling frames, and inclusion required survival and participation in the second resurvey. These selection processes may influence both generalizability and the observed associations. Sampling-source adjustment and baseline-disease exclusions cannot fully address these concerns. Medication information was incomplete, limiting assessment of lipid-lowering treatment, and recent weight loss before baseline could not be assessed. Finally, the fatty acid proportions are interdependent, and their associations depend on the compositional contrast being examined. The ω-6 finding was less consistent across alternative outcomes, while the log-ratio and subgroup results did not meet the expanded FDR criteria and remain exploratory.

## 5. Conclusions

This prospective study found that a higher circulating MUFA proportion was associated with a lower risk of excessive adiposity increase, whereas a higher ω-6 proportion was associated with a higher risk. These associations persisted after adjustment for sampling source and exclusion of baseline major diseases but were less consistent across alternative outcome definitions, particularly for ω-6. Further longitudinal studies using repeated, species-specific fatty acid measurements and detailed dietary assessments are needed to clarify the contributions of dietary exposure and endogenous fatty acid metabolism to these associations.

## Figures and Tables

**Table 1:** Baseline characteristics of the study population by BMI change.

Characteristic	All	BMI Increase <5%	BMI Increase ≥5%
No. of participants	1498	999	499
Demographic factors			
Age, mean (SD), years	47.9 (9.7)	49.0 (9.6)	45.7 (9.4)
Male, No. (%)	604 (40.3)	425 (42.5)	179 (35.9)
Urban residence, No. (%)	487 (32.5)	359 (35.9)	128 (25.7)
Socioeconomic factors			
Married, No. (%)	1427 (95.3)	954 (95.5)	473 (94.8)
Middle school and above, No. (%)	795 (53.1)	526 (52.7)	269 (53.9)
Household income ≥20,000 CNY/year, No. (%)	497 (33.2)	356 (35.6)	141 (28.3)
Lifestyle factors			
Current smoker, No. (%)	409 (27.3)	282 (28.2)	127 (25.5)
Weekly drinker, No. (%)	203 (13.6)	153 (15.3)	50 (10.0)
Physical activity, mean (SD), MET-h/d	22.9 (14.4)	22.2 (14.2)	24.3 (14.7)
Physical measurements			
Systolic blood pressure, mean (SD), mm Hg	129.9 (20.3)	131.5 (20.8)	126.7 (19.0)
Diastolic blood pressure, mean (SD), mm Hg	77.6 (11.2)	78.2 (11.2)	76.3 (11.1)
BMI, mean (SD), kg/m^2^	23.8 (3.3)	24.2 (3.4)	23.0 (3.1)
Waist circumference, mean (SD), cm	79.6 (9.5)	80.7 (9.6)	77.3 (9.0)
BMI change, mean (SD), %	2.4 (7.6)	−1.5 (5.1)	10.2 (5.2)
Waist circumference change, mean (SD), %	7.0 (8.6)	4.1 (7.4)	12.9 (7.7)

Values are unadjusted means (SDs) or numbers (percentages). Percentages use the nonmissing denominator for each variable. BMI, body mass index; CNY, Chinese yuan; WC, waist circumference; MET-h/d, metabolic equivalent of task hours per day; SD, standard deviation.

**Table 2:** Associations of circulating fatty acid proportions with baseline overweight or obesity and excessive adiposity increase.

Exposure	Baseline Overweight or Obesity			Excessive Adiposity Increase		
	PR (95% CI)	*p*	*q*	RR (95% CI)	*p*	*q*
SFA	0.965 (0.910, 1.024)	0.240	0.240	1.011 (0.936, 1.091)	0.785	0.785
MUFA	1.361 (1.287, 1.440)	<0.001	<0.001	0.870 (0.801, 0.945)	<0.001	0.004
ω-6	0.776 (0.735, 0.819)	<0.001	<0.001	1.115 (1.029, 1.208)	0.008	0.016
ω-3	1.117 (1.056, 1.181)	<0.001	<0.001	0.957 (0.886, 1.034)	0.268	0.357

Estimates are presented per 1-SD higher circulating fatty acid proportion. Modified Poisson regression with robust variance was used to estimate prevalence ratios (PRs) for baseline overweight or obesity and risk ratios (RRs) for excessive adiposity increase. Overweight or obesity was defined as BMI ≥ 24 kg/m^2^, and excessive adiposity increase was defined as a ≥5% increase in BMI from baseline. Models were adjusted for age, sex, the 10 CKB study regions, education, household income, marital status, smoking status, alcohol consumption, physical activity, and dietary frequencies. Models for excessive adiposity increase were additionally adjusted for baseline BMI. The Benjamini–Hochberg procedure was applied across the four fatty acid proportions separately within each outcome. BMI, body mass index; CI, confidence interval; FDR, false discovery rate; MUFA, monounsaturated fatty acids; PR, prevalence ratio; RR, risk ratio; SD, standard deviation; SFA, saturated fatty acids; ω-3, omega-3 polyunsaturated fatty acids; ω-6, omega-6 polyunsaturated fatty acids.

## Data Availability

The data underlying this study are available to bona fide researchers through the China Kadoorie Biobank Data Access System, subject to approval of a research proposal and completion of a Data Access Agreement. The authors are not permitted to share participant-level data directly.

## Supplementary Materials

The following supporting information can be downloaded at https://www.mdpi.com/article/10.3390/nu18203401/s1, Figure S1. Associations of circulating fatty acid proportions with waist circumference increase ≥5%; Figure S2. Associations of fatty acid log-ratio measures with BMI increase ≥5%; Table S1A. Participants by NMR sampling source; Table S1B. Nonexclusive study-selection categories; Table S2A. Standard deviations of circulating fatty acid proportions; Table S2B. Pearson correlations among fatty acid proportions; Table S2C. Spearman correlations among fatty acid proportions; Table S3A. Circulating fatty acid proportions and baseline-adjusted follow-up BMI; Table S3B. Circulating fatty acid proportions and absolute BMI change; Table S3C. Circulating fatty acid proportions and annualized BMI change; Table S3D. Overweight or obesity at follow-up among participants with baseline BMI < 24 kg/m^2^; Table S4A. Sensitivity analysis using BMI increase ≥3%; Table S4B. Sensitivity analysis using BMI increase ≥10%; Table S4C. BMI increase ≥5% after excluding participants selected as disease cases; Table S4D. BMI increase ≥5% after excluding baseline major diseases; Table S4E. BMI increase ≥5% after additional adjustment for sampling source; Table S4F. BMI increase ≥5% within NMR sampling-source groups; Table S5. Baseline major diseases and statin-use information; Table S6. Families of tests used for FDR correction; Table S7A. Interaction tests for BMI increase ≥5%; Table S7B. Participants and events in the BMI subgroups shown in Figure 2; Table S8A. Pairwise fatty acid reallocation and BMI increase ≥5%; Table S8B. Pairwise fatty acid reallocation and follow-up BMI; Table S9. FDR correction for the BMI log-ratio analyses.

## Abbreviations

The following abbreviations are used in this manuscript:

ANCOVA	Analysis of covariance
BMI	Body mass index
CI	Confidence interval
CKB	China Kadoorie Biobank
CNY	Chinese yuan
DHA	Docosahexaenoic acid
FDR	False discovery rate
HC3	Heteroscedasticity-consistent type 3 covariance estimator
MET-h/d	Metabolic equivalent of task hours per day
MUFA	Monounsaturated fatty acids
NMR	Nuclear magnetic resonance
PR	Prevalence ratio
PUFA	Polyunsaturated fatty acids
RR	Risk ratio
SD	Standard deviation
SFA	Saturated fatty acids
WC	Waist circumference
