# Supplementary material for: Circulating Fatty Acid Composition and Subsequent Adiposity Gain: A Prospective Study in the China Kadoorie Biobank
Source: Nutrients. 2026 Oct 16;18(20):3401. doi: 10.3390/nu18203401 (PMC13708298; doi:10.3390/nu18203401)
Supplement: Supplementary file 1 [file nutrients-18-03401-s001.zip › nutrients-4595085-supplementary.pdf]

Figure S1. Associations of circulating fatty acid proportions with waist circumference increase ≥5%

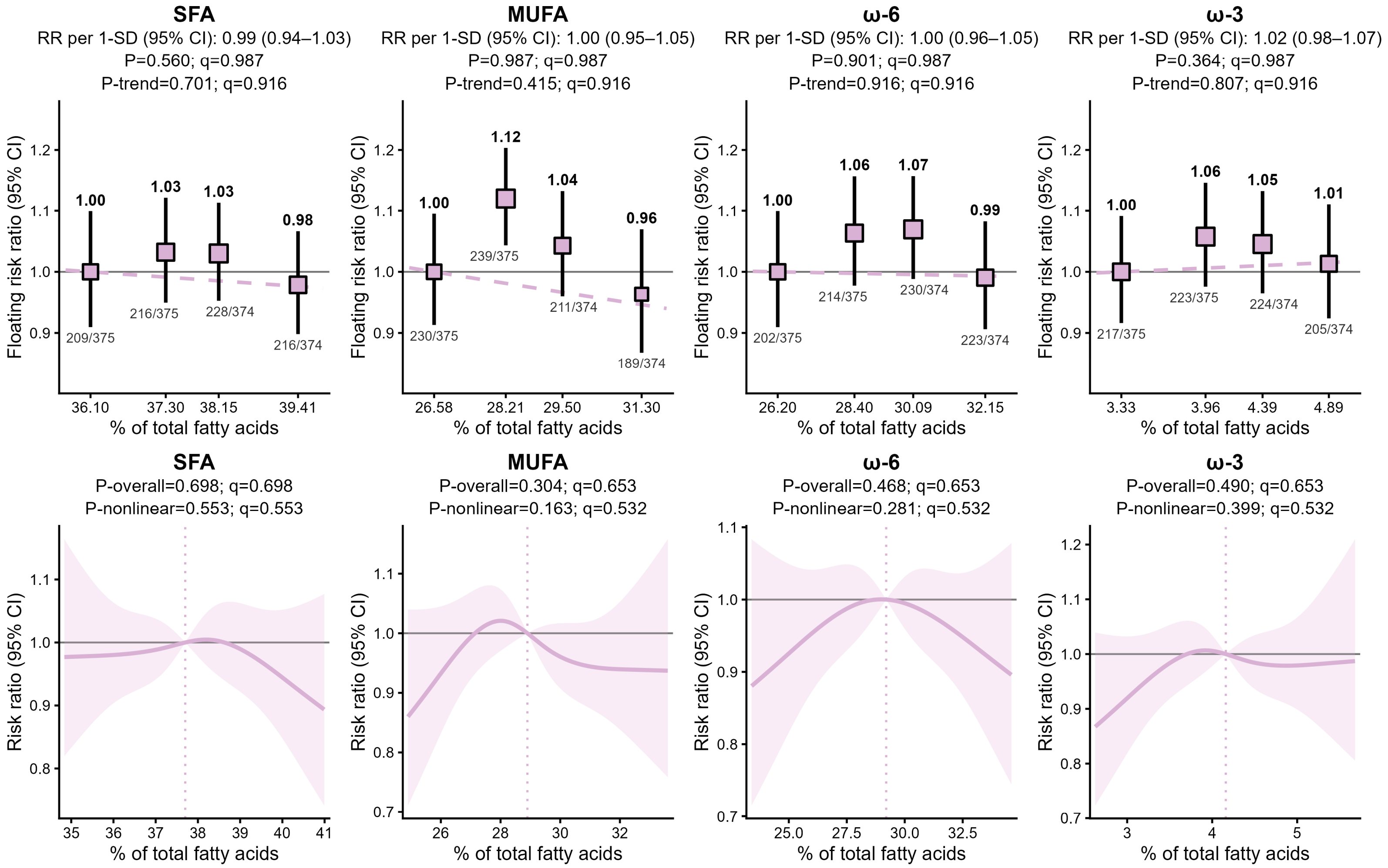

The upper panels show quartile-specific floating risk ratios with 95% confidence intervals; labels below the estimates indicate events/participants in each quartile. The dashed lines show the fitted quartile-trend associations. The lower panels show restricted cubic spline associations, with shaded areas indicating 95% confidence intervals. Models were adjusted for age, sex, the 10 CKB study regions, education, household income, marital status, smoking status, alcohol consumption, physical activity, dietary frequencies, and baseline waist circumference. Benjamini–Hochberg correction was applied across the four fatty acid classes separately for each test type. SFA, saturated fatty acids; MUFA, monounsaturated fatty acids; RR, risk ratio; SD, standard deviation; CI, confidence interval; *q*, FDR-adjusted *P* value.

Figure S2. Associations of fatty acid log-ratio measures with BMI increase ≥5%

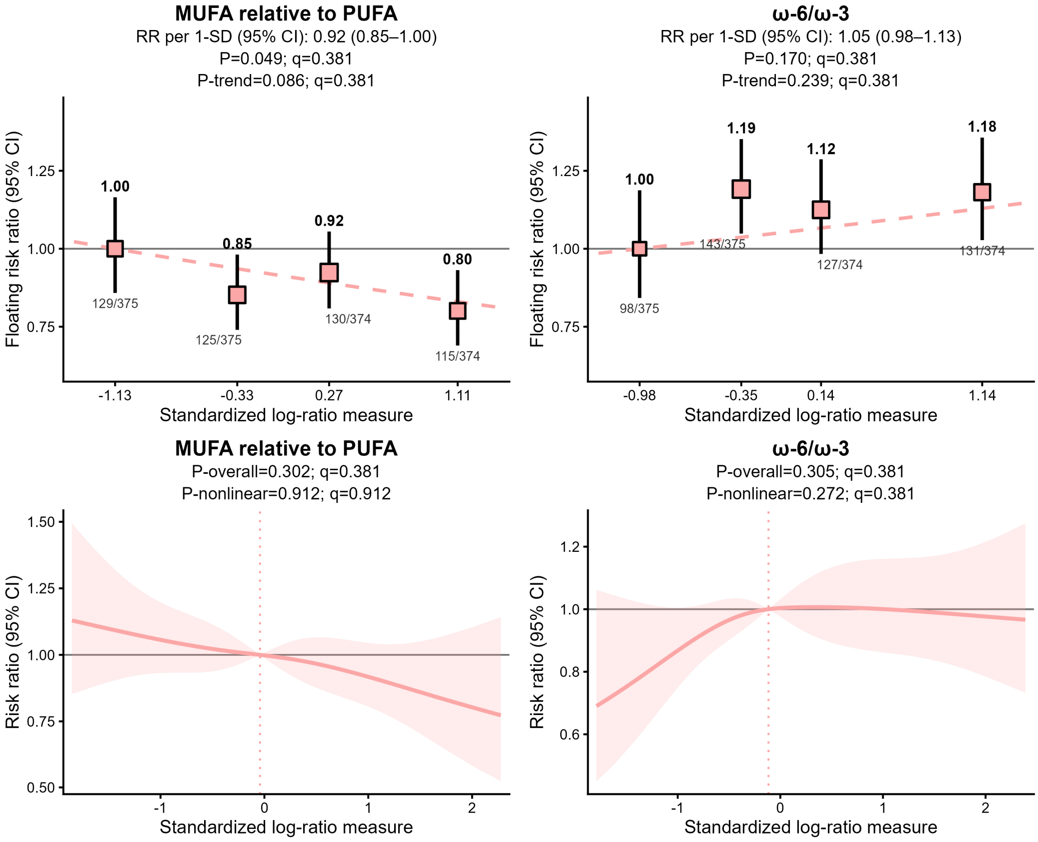

The upper panels show quartile-specific floating risk ratios with 95% confidence intervals; labels below the estimates indicate events/participants in each quartile. The dashed lines show the fitted quartile-trend associations. The lower panels show restricted cubic spline associations, with shaded areas indicating 95% confidence intervals. The MUFA-relative-to-PUFA measure was calculated as √(2/3) × ln[MUFA/√(ω-6 × ω-3)], and the ω-6-relative-to-ω-3 measure as √(1/2) × ln(ω-6/ω-3). Both measures were standardized. Models included the original covariates, baseline BMI, and log-transformed total fatty acid concentration. The q values are based on joint Benjamini–Hochberg correction across the ten BMI log-ratio tests reported in Table S9. BMI, body mass index; MUFA, monounsaturated fatty acids; PUFA, polyunsaturated fatty acids; RR, risk ratio; SD, standard deviation; CI, confidence interval; *q*, FDR-adjusted *P* value.

Table S1 (A). Participants by NMR sampling source

Sampling source	N
Both study sources	186
Cardiovascular disease studies only	898
Pancreatic cancer or diabetes studies only	414

The categories are mutually exclusive. Participants selected through both sources were included once in the analytic sample. CVD, cardiovascular disease; NMR, nuclear magnetic resonance.

Table S1 (B). Nonexclusive study-selection categories

Selection category	N selected
CVD studies: COPD exacerbation cases	0
CVD studies: fatal ischaemic heart disease cases	0
CVD studies: intracerebral haemorrhage cases	33
CVD studies: ischaemic stroke cases	69
CVD studies: myocardial infarction cases	18
CVD studies: subarachnoid haemorrhage cases	<5
CVD studies: controls for intracerebral haemorrhage	814
CVD studies: second-resurvey selection	148
CVD studies: unselected category	<5
Pancreatic cancer/diabetes studies: diabetes cases	37
Pancreatic cancer/diabetes studies: pancreatic cancer cases	6
Pancreatic cancer/diabetes studies: subcohort	559

These study-selection categories can overlap and should not be summed to obtain the analytic sample size. Case selection in the contributing studies is distinct from disease history at baseline. Counts below 5 are displayed as <5. COPD, chronic obstructive pulmonary disease.

Table S2 (A). Standard deviations of circulating fatty acid proportions

Fatty acid	N	SD percentage points
SFA	1498	1.4994
MUFA	1498	2.0732
ω-6	1498	2.6886
ω-3	1498	0.7221

SDs are expressed in percentage points. Each fatty acid proportion was standardized using its mean and SD in the full analytic sample. SD, standard deviation.

Table S2 (B). Pearson correlations among fatty acid proportions

Fatty acid	SFA	MUFA	ω-6	ω-3
SFA	—	0.035 (*q*=0.178)	-0.564 (*q*<0.001)	-0.074 (*q*=0.007)
MUFA	0.035 (*q*=0.178)	—	-0.800 (*q*<0.001)	0.041 (*q*=0.136)
ω-6	-0.564 (*q*<0.001)	-0.800 (*q*<0.001)	—	-0.258 (*q*<0.001)
ω-3	-0.074 (*q*=0.007)	0.041 (*q*=0.136)	-0.258 (*q*<0.001)	—

Cells show the correlation coefficient followed by the FDR-adjusted *q* value in parentheses. Benjamini–Hochberg correction was applied to the six unique pairwise tests separately for each correlation method. Diagonal values are omitted.

Table S2 (C). Spearman correlations among fatty acid proportions

Fatty acid	SFA	MUFA	ω-6	ω-3
SFA	—	0.035 (*q*=0.208)	-0.538 (*q*<0.001)	-0.079 (*q*=0.003)
MUFA	0.035 (*q*=0.208)	—	-0.793 (*q*<0.001)	0.033 (*q*=0.208)
ω-6	-0.538 (*q*<0.001)	-0.793 (*q*<0.001)	—	-0.224 (*q*<0.001)
ω-3	-0.079 (*q*=0.003)	0.033 (*q*=0.208)	-0.224 (*q*<0.001)	—

Cells show the correlation coefficient followed by the FDR-adjusted *q* value in parentheses. Benjamini–Hochberg correction was applied to the six unique pairwise tests separately for each correlation method. Diagonal values are omitted.

Table S3 (A). Circulating fatty acid proportions and baseline-adjusted follow-up BMI

Fatty acid	N	β (95% CI)	*P*	*q*
SFA	1498	0.048 (-0.053, 0.148)	0.353	0.470
MUFA	1498	-0.119 (-0.221, -0.018)	0.022	0.086
ω-6	1498	0.070 (-0.035, 0.175)	0.191	0.383
ω-3	1498	-0.032 (-0.126, 0.063)	0.509	0.509

β coefficients and 95% CIs represent adjusted mean differences in follow-up BMI (kg/m^2) per 1-SD higher fatty acid proportion. Linear models used HC3 robust standard errors. Models were adjusted for baseline BMI, age, sex, the 10 CKB study regions, education, household income, marital status, smoking, alcohol consumption, physical activity and dietary frequencies. Benjamini–Hochberg correction was applied across the four fatty acid classes.

Table S3 (B). Circulating fatty acid proportions and absolute BMI change

Fatty acid	N	β (95% CI)	*P*	*q*
SFA	1498	0.048 (-0.053, 0.148)	0.353	0.470
MUFA	1498	-0.119 (-0.221, -0.018)	0.022	0.086
ω-6	1498	0.070 (-0.035, 0.175)	0.191	0.383
ω-3	1498	-0.032 (-0.126, 0.063)	0.509	0.509

β coefficients and 95% CIs are in kg/m^2 per 1-SD higher fatty acid proportion. Linear models used HC3 robust standard errors. Models were adjusted for baseline BMI, age, sex, the 10 CKB study regions, education, household income, marital status, smoking, alcohol consumption, physical activity and dietary frequencies. Benjamini–Hochberg correction was applied across the four fatty acid classes. With the same participants and baseline adjustment, absolute-change and follow-up BMI models yield the same exposure coefficients and confidence intervals.

Table S3 (C). Circulating fatty acid proportions and annualized BMI change

Fatty acid	N	β (95% CI)	*P*	*q*
SFA	1498	0.006 (-0.007, 0.018)	0.374	0.427
MUFA	1498	-0.014 (-0.027, -0.002)	0.027	0.107
ω-6	1498	0.009 (-0.004, 0.021)	0.194	0.387
ω-3	1498	-0.005 (-0.016, 0.007)	0.427	0.427

β coefficients and 95% CIs are in kg/m^2/year per 1-SD higher fatty acid proportion. Linear models used HC3 robust standard errors. Models were adjusted for baseline BMI, age, sex, the 10 CKB study regions, education, household income, marital status, smoking, alcohol consumption, physical activity and dietary frequencies. Annualized change was absolute BMI change divided by the interval between measurements in years. Benjamini–Hochberg correction was applied across the four fatty acid classes.

Table S3 (D). Overweight or obesity at follow-up among participants with baseline BMI <24 kg/m²

Fatty acid	N	Events	RR (95% CI)	*P*	*q*
SFA	799	164	1.157 (1.008, 1.327)	0.038	0.153
MUFA	799	164	1.011 (0.885, 1.156)	0.869	0.869
ω-6	799	164	0.914 (0.795, 1.050)	0.205	0.410
ω-3	799	164	0.977 (0.836, 1.141)	0.766	0.869

The outcome was follow-up BMI ≥24 kg/m² among participants with baseline BMI <24 kg/m². RRs and 95% CIs are per 1-SD higher fatty acid proportion and were estimated using modified Poisson regression with robust variance. Models were adjusted for baseline BMI, age, sex, the 10 CKB study regions, education, household income, marital status, smoking, alcohol consumption, physical activity and dietary frequencies. Benjamini–Hochberg correction was applied across the four fatty acid classes within each analysis. Models additionally adjusted for the interval between measurements.

Table S4 (A). Sensitivity analysis using BMI increase ≥3%

Fatty acid	N	Events	RR (95% CI)	*P*	*q*
SFA	1498	651	1.024 (0.964, 1.088)	0.437	0.437
MUFA	1498	651	0.919 (0.862, 0.981)	0.011	0.044
ω-6	1498	651	1.061 (0.996, 1.132)	0.067	0.134
ω-3	1498	651	0.958 (0.900, 1.019)	0.176	0.235

The outcome was BMI increase ≥3%. RRs and 95% CIs are per 1-SD higher fatty acid proportion and were estimated using modified Poisson regression with robust variance. Models were adjusted for baseline BMI, age, sex, the 10 CKB study regions, education, household income, marital status, smoking, alcohol consumption, physical activity and dietary frequencies. Benjamini–Hochberg correction was applied across the four fatty acid classes within each analysis. For comparison, the primary BMI increase ≥5% endpoint occurred in 499 of 1498 participants.

Table S4 (B). Sensitivity analysis using BMI increase ≥10%

Fatty acid	N	Events	RR (95% CI)	*P*	*q*
SFA	1498	203	1.069 (0.931, 1.227)	0.346	0.761
MUFA	1498	203	0.977 (0.843, 1.131)	0.753	0.761
ω-6	1498	203	0.971 (0.842, 1.120)	0.689	0.761
ω-3	1498	203	1.022 (0.889, 1.175)	0.761	0.761

The outcome was BMI increase ≥10%. RRs and 95% CIs are per 1-SD higher fatty acid proportion and were estimated using modified Poisson regression with robust variance. Models were adjusted for baseline BMI, age, sex, the 10 CKB study regions, education, household income, marital status, smoking, alcohol consumption, physical activity and dietary frequencies. Benjamini–Hochberg correction was applied across the four fatty acid classes within each analysis.

Table S4 (C). BMI increase ≥5% after excluding participants selected as disease cases

Fatty acid	N	Events	RR (95% CI)	*P*	*q*
SFA	1334	446	0.997 (0.919, 1.082)	0.946	0.946
MUFA	1334	446	0.879 (0.806, 0.960)	0.004	0.016
ω-6	1334	446	1.121 (1.030, 1.219)	0.008	0.016
ω-3	1334	446	0.937 (0.863, 1.017)	0.119	0.158

Participants selected as disease cases in the contributing NMR studies were excluded. RRs and 95% CIs are per 1-SD higher fatty acid proportion and were estimated using modified Poisson regression with robust variance. Models were adjusted for baseline BMI, age, sex, the 10 CKB study regions, education, household income, marital status, smoking, alcohol consumption, physical activity and dietary frequencies. Benjamini–Hochberg correction was applied across the four fatty acid classes within each analysis. This exclusion concerns study-selection status, rather than disease history at baseline.

Table S4 (D). BMI increase ≥5% after excluding baseline major diseases

Fatty acid	N	Events	RR (95% CI)	*P*	*q*
SFA	1410	486	0.998 (0.924, 1.079)	0.969	0.969
MUFA	1410	486	0.872 (0.802, 0.948)	0.001	0.005
ω-6	1410	486	1.121 (1.033, 1.216)	0.006	0.012
ω-3	1410	486	0.958 (0.886, 1.036)	0.286	0.381

RRs and 95% CIs are per 1-SD higher fatty acid proportion and were estimated using modified Poisson regression with robust variance. Models were adjusted for baseline BMI, age, sex, the 10 CKB study regions, education, household income, marital status, smoking, alcohol consumption, physical activity and dietary frequencies. Benjamini–Hochberg correction was applied across the four fatty acid classes within each analysis. Participants with baseline diabetes, chronic heart disease, stroke/TIA or cancer were excluded. Participants with unknown required disease status were not classified as disease-free.

Table S4 (E). BMI increase ≥5% after additional adjustment for sampling source

Fatty acid	N	Events	RR (95% CI)	*P*	*q*
SFA	1498	499	1.012 (0.937, 1.092)	0.768	0.768
MUFA	1498	499	0.864 (0.792, 0.942)	<0.001	0.004
ω-6	1498	499	1.128 (1.033, 1.232)	0.007	0.014
ω-3	1498	499	0.954 (0.874, 1.041)	0.292	0.389

The outcome was BMI increase ≥5%. RRs and 95% CIs are per 1-SD higher fatty acid proportion and were estimated using modified Poisson regression with robust variance. Models were adjusted for baseline BMI, age, sex, the 10 CKB study regions, education, household income, marital status, smoking, alcohol consumption, physical activity and dietary frequencies. Benjamini–Hochberg correction was applied across the four fatty acid classes within each analysis. The adjusted model additionally included sampling source. These models did not use sampling weights.

Table S4 (F). BMI increase ≥5% within NMR sampling-source groups

Analysis	Fatty acid	N	Events	RR (95% CI)	*P*	*q*
Both study sources	SFA	186	50	0.804 (0.623, 1.038)	0.094	0.374
	MUFA	186	50	0.870 (0.615, 1.231)	0.433	0.546
	ω-6	186	50	1.181 (0.853, 1.636)	0.316	0.546
	ω-3	186	50	1.141 (0.744, 1.749)	0.546	0.546
Cardiovascular disease studies only	SFA	898	325	1.019 (0.926, 1.121)	0.706	0.706
	MUFA	898	325	0.848 (0.760, 0.946)	0.003	0.012
	ω-6	898	325	1.150 (1.022, 1.293)	0.020	0.039
	ω-3	898	325	0.945 (0.832, 1.073)	0.382	0.509
Pancreatic cancer or diabetes studies only	SFA	414	124	1.025 (0.882, 1.192)	0.743	0.743
	MUFA	414	124	0.819 (0.688, 0.975)	0.025	0.099
	ω-6	414	124	1.157 (0.987, 1.355)	0.071	0.143
	ω-3	414	124	0.929 (0.782, 1.103)	0.400	0.534

The outcome was BMI increase ≥5%. RRs and 95% CIs are per 1-SD higher fatty acid proportion and were estimated using modified Poisson regression with robust variance. Models were adjusted for baseline BMI, age, sex, the 10 CKB study regions, education, household income, marital status, smoking, alcohol consumption, physical activity and dietary frequencies. Benjamini–Hochberg correction was applied across the four fatty acid classes within each analysis. Stratified models omitted covariates constant within a stratum. These models did not use sampling weights.

Table S5. Baseline major diseases and statin-use information

Characteristic	Yes, n	Available N	Yes, %	Unavailable N
Diabetes (has_diabetes definition)	61	1498	4.1	0
Self-reported chronic heart disease	17	1498	1.1	0
Self-reported stroke or TIA	11	1498	0.7	0
Self-reported cancer	<5	1498	0.2	0
Current statin use	<5	120	1.7	1378
Any baseline major disease	88	1498	5.9	0

Diabetes was defined as self-reported physician-diagnosed diabetes or screen-detected diabetes (fasting blood glucose ≥7.0 mmol/L or random blood glucose ≥11.1 mmol/L). Other diseases were based on self-reported diagnoses. Major disease comprised diabetes, chronic heart disease, stroke/TIA or cancer. Percentages use the available N for each characteristic. Statin information was incomplete and was not a covariate in the primary models; unavailable status was not classified as non-use. TIA, transient ischaemic attack.

Table S6. Families of tests used for FDR correction

Analysis	Tests per family	Correction family
Per-SD fatty acid associations	4	Four fatty acid classes; separately for baseline overweight/obesity, BMI increase ≥5% and WC increase ≥5%
Quartile trends	4	Four fatty acid classes, separately for each adiposity outcome
Spline overall tests	4	Four fatty acid classes, separately for each adiposity outcome
Spline nonlinearity tests	4	Four fatty acid classes, separately for each adiposity outcome
Continuous follow-up BMI	4	Four fatty acid classes
Alternative BMI outcomes and sensitivity analyses	4	Four fatty acid classes within each analysis and outcome definition
Pairwise reallocation	6	Six pairwise reallocation contrasts, separately for BMI increase ≥5% and continuous follow-up BMI
BMI subgroup interactions	36	Four fatty acid classes × nine subgroup variables, jointly
BMI log-ratio analyses	10	Two measures × five test types, jointly: binary per-SD, quartile trend, spline overall, spline nonlinearity and continuous follow-up per-SD
Fatty acid correlations	6	Six unique pairs, separately for Pearson and Spearman correlations

All listed families used the Benjamini–Hochberg procedure. *q* values are FDR-adjusted P values; the reported 95% confidence intervals were not adjusted for multiple testing. FDR, false discovery rate.

Table S7 (A). Interaction tests for BMI increase ≥5%

Fatty acid	Subgroup variable	Interaction *P*	*q*
SFA	Age, years	0.851	0.940
SFA	Sex	0.332	0.714
SFA	Area type	0.981	0.985
SFA	Education	0.140	0.714
SFA	Household income, CNY/year	0.011	0.233
SFA	Current smoking	0.738	0.916
SFA	Weekly alcohol consumption	0.856	0.940
SFA	Physical activity, MET-h/d	0.369	0.714
SFA	Baseline BMI, kg/m²	0.302	0.714
MUFA	Age, years	0.230	0.714
MUFA	Sex	0.556	0.769
MUFA	Area type	0.086	0.606
MUFA	Education	0.019	0.233
MUFA	Household income, CNY/year	0.548	0.769
MUFA	Current smoking	0.495	0.743
MUFA	Weekly alcohol consumption	0.417	0.714
MUFA	Physical activity, MET-h/d	0.247	0.714
MUFA	Baseline BMI, kg/m²	0.055	0.498
ω-6	Age, years	0.287	0.714
ω-6	Sex	0.271	0.714
ω-6	Area type	0.101	0.606
ω-6	Education	0.013	0.233
ω-6	Household income, CNY/year	0.609	0.812
ω-6	Current smoking	0.379	0.714
ω-6	Weekly alcohol consumption	0.322	0.714
ω-6	Physical activity, MET-h/d	0.488	0.743
ω-6	Baseline BMI, kg/m²	0.450	0.736
ω-3	Age, years	0.330	0.714
ω-3	Sex	0.890	0.942
ω-3	Area type	0.695	0.894
ω-3	Education	0.985	0.985
ω-3	Household income, CNY/year	0.342	0.714
ω-3	Current smoking	0.766	0.919
ω-3	Weekly alcohol consumption	0.401	0.714
ω-3	Physical activity, MET-h/d	0.235	0.714
ω-3	Baseline BMI, kg/m²	0.862	0.940

Interaction tests were based on multiplicative terms between each standardized fatty acid proportion and each subgroup variable. Benjamini–Hochberg correction was applied jointly to 36 tests (four fatty acid classes × nine subgroup variables).

Table S7 (B). Participants and events in the BMI subgroups shown in Figure 2

Subgroup variable	Stratum	Participants, N	Events, n
Age, years	<45.6	749	310
Age, years	≥45.6	749	189
Sex	Male	604	179
Sex	Female	894	320
Area type	Rural	1011	371
Area type	Urban	487	128
Education	Primary school or below	703	230
Education	Middle school or above	795	269
Household income, CNY/year	<20,000	1001	358
Household income, CNY/year	≥20,000	497	141
Current smoking	No	1089	372
Current smoking	Yes	409	127
Weekly alcohol consumption	No	1295	449
Weekly alcohol consumption	Yes	203	50
Physical activity, MET-h/d	<19.3	748	224
Physical activity, MET-h/d	≥19.3	750	275
Baseline BMI, kg/m²	<24	799	323
Baseline BMI, kg/m²	≥24	699	176

Events were defined as a BMI increase of ≥5% from baseline. Counts refer to participants included in the corresponding subgroup models and are the same for the MUFA and ω-6 analyses in Figure 2. Within each subgroup variable, the two strata together comprise 1,498 participants and 499 events. BMI, body mass index; MUFA, monounsaturated fatty acids; CNY, Chinese yuan; MET-h/d, metabolic equivalent of task hours per day.

Table S8 (A). Pairwise fatty acid reallocation and BMI increase ≥5%

Fatty acid replacement	N	RR (95% CI)	*P*	*q*
MUFA to ω-3	1498	1.033 (0.919, 1.160)	0.588	0.826
SFA to MUFA	1498	0.955 (0.890, 1.025)	0.205	0.616
SFA to ω-3	1498	0.986 (0.874, 1.114)	0.826	0.826
SFA to ω-6	1498	1.009 (0.956, 1.065)	0.752	0.826
ω-6 to MUFA	1498	0.947 (0.909, 0.986)	0.009	0.052
ω-6 to ω-3	1498	0.978 (0.879, 1.088)	0.683	0.826

The four fatty acid proportions were normalized to sum to 100%. Each contrast represents a 1-percentage-point decrease in the proportion of the replaced fatty acid accompanied by an equal increase in the proportion of the replacement fatty acid, holding the other two proportions constant. The proportion of the replaced fatty acid was omitted from the model; the other three proportions, baseline BMI, standardized log-transformed total fatty acid concentration, and the original covariates were included. Modified Poisson regression with robust variance was used to estimate RRs. Benjamini–Hochberg correction was applied across the six contrasts.

Table S8 (B). Pairwise fatty acid reallocation and follow-up BMI

Fatty acid replacement	N	β (95% CI)	*P*	*q*
MUFA to ω-3	1498	0.034 (-0.108, 0.177)	0.638	0.943
SFA to MUFA	1498	-0.053 (-0.140, 0.034)	0.234	0.702
SFA to ω-3	1498	-0.018 (-0.166, 0.129)	0.807	0.943
SFA to ω-6	1498	-0.014 (-0.082, 0.055)	0.696	0.943
ω-6 to MUFA	1498	-0.039 (-0.092, 0.014)	0.147	0.702
ω-6 to ω-3	1498	-0.005 (-0.137, 0.128)	0.943	0.943

The four fatty acid proportions were normalized to sum to 100%. Each contrast represents a 1-percentage-point decrease in the proportion of the replaced fatty acid accompanied by an equal increase in the proportion of the replacement fatty acid, holding the other two proportions constant. The proportion of the replaced fatty acid was omitted from the model; the other three proportions, baseline BMI, standardized log-transformed total fatty acid concentration, and the original covariates were included. Linear models used HC3 robust standard errors; estimates are expressed in kg/m^2. Benjamini–Hochberg correction was applied across the six contrasts.

Table S9. FDR correction for the BMI log-ratio analyses

Log-ratio measure	Test	***P***	***q***
MUFA relative to PUFA	Binary per-SD	0.049	0.381
ω-6 relative to ω-3	Binary per-SD	0.170	0.381
MUFA relative to PUFA	Continuous follow-up per-SD	0.262	0.381
ω-6 relative to ω-3	Continuous follow-up per-SD	0.702	0.780
MUFA relative to PUFA	Quartile trend	0.086	0.381
ω-6 relative to ω-3	Quartile trend	0.239	0.381
MUFA relative to PUFA	Spline overall	0.302	0.381
ω-6 relative to ω-3	Spline overall	0.305	0.381
MUFA relative to PUFA	Spline nonlinearity	0.912	0.912
ω-6 relative to ω-3	Spline nonlinearity	0.272	0.381

The MUFA-relative-to-PUFA log-ratio was $\sqrt{(2/3)} \times \ln[\mathrm{MUFA}/\sqrt{(\omega\text{-}6 \times \omega\text{-}3)}]$; the ω-6-relative-to-ω-3 log-ratio was $\sqrt{(1/2)} \times \ln(\omega\text{-}6/\omega\text{-}3)$. Both were standardized to 1 SD. Models included baseline BMI, log total fatty acid concentration and the original covariates. Benjamini–Hochberg correction was applied jointly to the ten tests shown.
